# Cognitive and Neuropsychiatric Features of COVID-19 Patients After Hospital Dismission: An Italian Sample

**DOI:** 10.3389/fpsyg.2022.908363

**Published:** 2022-05-24

**Authors:** Veronica Cian, Alessandro De Laurenzis, Chiara Siri, Anna Gusmeroli, Margherita Canesi

**Affiliations:** ^1^Parkinson’s Disease Department, Moriggia-Pelascini Hospital, Gravedona ed Uniti, Italy; ^2^Movement Disorders Rehabilitation Department, Moriggia-Pelascini Hospital, Gravedona ed Uniti, Italy

**Keywords:** cognition, teleneuropsychology, memory, executive functions, COVID-19

## Abstract

**Background and Aims:**

Recent studies suggest cognitive, emotional, and behavioral impairments occur in patients after SARS-CoV-2 infection. However, studies are limited to case reports or case series and, to our knowledge, few of them have control groups. This study aims to assess the prevalence of neuropsychological and neuropsychiatric impairment in patients after hospitalization.

**Methods:**

We enrolled 29 COVID+ patients (M/F: 17/12; age 58.41 ± 10.00 years; education 11.07 ± 3.77 years, 2 left handers) who needed hospitalization but no IC, about 20 days post-dismission, and 29 COVID− healthy matched controls. Neuropsychological and neuropsychiatric assessments were conducted *via* teleneuropsychology using the following tests: MMSE, CPM47, RAVLT, CDT, Digit-Span Forward/Backward, Verbal fluencies; BDI-II, STAI. People with previous reported cognitive impairment and neurological or psychiatric conditions were excluded. Clinical and demographics were collected. Comparison between groups was conducted using parametric or non-parametric tests according to data distribution (*T*-test, Mann Withney-*U* test; Chi-square goodness of fit). Within COVID+ group, we also evaluated the correlation between the cognitive and behavioral assessment scores and clinical variables collected.

**Results:**

Among COVID+, 62% had at least one pathological test (vs. 13% in COVID−; *p* = 0.000) and significantly worst performances than COVID− in RAVLT learning (42.55 ± 10.44 vs. 47.9 ± 8.29, *p* = 0.035), RAVLT recall (8.79 ± 3.13 vs. 10.38 ± 2.19, *p* = 0.03), and recognition (13.69 ± 1.47 vs. 14.52 ± 0.63, *p* = 0.07). STAI II was higher in COVID− (32.69 ± 7.66 vs. 39.14 ± 7.7, *p* = 0.002). Chi-square on dichotomous values (normal/pathological) showed a significant difference between groups in Digit backward test (pathological 7/29 COVID+ vs. 0/29 COVID−; *p* = 0.005).

**Conclusions:**

Patients COVID+ assessed by teleneuropsychology showed a vulnerability in some memory and executive functions (working memory, learning, delayed recall, and recognition). Intriguingly, anxiety was higher in the control group. Our findings therefore confirm the impact of COVID-19 on cognition even in patients who did not need IC. Follow-up is needed to evaluate the evolution of COVID-19-related cognitive deficit.

**Clinical Trial Registration:**

[ClinicalTrials.gov], identifier [NCT05143320].

## Introduction

SARS-CoV-2 is the agent of the current COVID-19 pandemic; it may be responsible for different and various clinical features, ranging from asymptomatic to critical health conditions.

The main symptoms include fever, fatigue, dyspnea, and, in the most serious cases, respiratory failure and consequent hospitalization. There are different clinical features depending on the severity of infection: mild with no dyspnea and normal level of blood oxygen saturation (SatO2); moderate with dyspnea and SatO2 = 94–98% and radiological signs of pneumonia; severe, with dyspnea, SatO2 < 93%, respiratory rate (RR) > 30/min, radiological progression of lesions with O2 supplementation required, eventually with non-invasive ventilation; and critical with patients needing mechanical ventilation (Carda et al., 2020; Alemanno et al., 2021). In this contest, patients can present multisystemic complications, in particular in cardiorespiratory and neurological systems.

In fact, recent studies have highlighted the repercussions of SARS-CoV-2 on the Central Nervous System (CNS) and related neurological (Ferrarese et al., 2020; Khatoon et al., 2020). Migraine, dizziness, anosmia, dysgeusia, stroke, Guillain–Barré syndrome, and FAS (Foreign Accent Syndrome) were found, probably due to the neuroinvasive nature of the virus (Cotelli et al., 2020). The neuronal pathway of infection *via* the olfactory nerve and role of ACE2 has been observed to be the principal pathophysiological mechanisms contributing to neuropsychiatric and cognitive impairments in COVID-19 (Mirfazeli et al., 2020; Pantelis et al., 2020; Kumar and Nayar, 2021). Therefore, it is assumed that neuropsychiatric symptoms may occur as a result of a proinflammatory response in the CNS. Another way of entry of the virus is through peripheral myeloid cells infected by SARS-CoV-2 and the transmigration to the blood–brain barrier *via* peripheral immune cells (Yesilkaya and Balcioglu, 2020).

Multiple factors related to the illness and its treatment such as delirium, cerebrovascular events, inflammation, hypoxia, ventilation, and sedation can contribute to cognitive impairment development (Jaywant et al., 2021).

Acute respiratory distress syndrome (ARDS) is known to carry an increased risk of developing deficits in cognitive functions with a prevalence of about 20% in 5 years after the disease (Levin et al., 2020; Riordan et al., 2020). Problems with memory, attention, information processing, and executive functions are particularly prevalent in these patients, probably due to hypothesized sensitivity of the hippocampus to the virus (Wilson B. A. et al., 2020). These impairments in cognitive functions are related to the level of severity of infection (PCR value) (Almeria et al., 2020; Zhou et al., 2020). Moreover, as pointed out in the study by Yesilkaya et al. (2021), cognitive functions are related to glutamatergic activity in the DLPFC and hippocampus, therefore damage or neurotransmitter dysfunctions in these brain regions will likely result in cognitive dysfunction.

Cognitive impairment is also present in patients with no neurological, neuropsychological, and neuropsychiatric history (Beaud et al., 2021). In these patients, a diagnosis of COVID-19 was associated with increased incidence of a first psychiatric diagnosis in the following 14–90 days compared with other health events (Taquet et al., 2021). Delirium, insomnia, anxiety, and depression reported during hospitalization persist even after complete remission (Rogers et al., 2020).

Studies investigating cognitive functioning in patients who had COVID-19 are limited to case reports or case series. Few investigations used objective neuropsychological measures to quantify cognitive deficits, or to describe the profile of cognitive dysfunction during recovery from COVID-19. In addition, few studies used a healthy control group to compare the cognitive and neuropsychiatric performance of patients with COVID-19. Recently, an Italian study compared neuropsychological sequelae in health care workers affected by COVID-19 4 months after the diagnosis with health care workers who were not affected by the virus; anxiety, stress, and depression scores are significantly higher in COVID-19 than in controls, but significant differences in cognitive functioning were not reported (Mattioli et al., 2020).

As concerns neuropsychological assessment, raising evidence shows that cognitive assessment delivered *via* telehealth is a reliable instrument. Recently, Marra et al. (2020) showed that, in response to the COVID-19 pandemic, some neuropsychological tests administered generally in paper-pencil versions have a good validity in identifying possible cognitive dysfunctions even if administered by videoconference. Consequently, these methods may represent a valid alternative to be used frequently for assessing cognitive functioning in older adults (Sozzi et al., 2020; Bilder et al., 2021).

The aim of the present study is to describe neuropsychological and neuropsychiatric features in patients recovered from moderate to severe forms of COVID-19 some weeks after hospital dismission.

In particular, we focused on the onset of these symptoms after few weeks in order to exclude that these difficulties are related to the period of hospitalization and the consequent environmental deprivation of the patients during admission in the COVID ward. Moreover, it is of great scientific and clinical relevance to describe COVID-19-related cognitive symptoms due to its possible reversibility.

## Materials and Methods

This is a prospective, observational single-center study, conducted at the Department of Parkinson’s disease and Movement disorders Rehabilitation of the “Moriggia-Pelascini” Hospital (Gravedona ed Uniti, Italy).

### Subjects

We asked to take part in the study to 45 people who were hospitalized in the COVID ward and recovered from the acute phase of COVID-19 (COVID+) with no need for ICU admission. All patients were hospitalized at the COVID-19 department, “Moriggia-Pelascini” Hospital [Gravedona ed Uniti (CO), Italy] between January and May 2021.

Inclusion criteria were: SARS-CoV-2 infection confirmed by positive PCR from nasopharyngeal swab; previous hospitalization in the COVID ward with no need of ICU admission; consent to participate in the study.

The general exclusion criteria for all participants included: age > 70 years; diagnosis of clinically relevant psychiatric disorders, psychosis, and/or delirium; diagnosis of neurological diseases; previous cognitive impairment subjective or reported by caregiver; any focal brain lesion detected with brain imaging studies (CT or MRI); hearing or visual impairments that may interfere with assessment; other medical conditions negatively affecting the cognitive status.

In the COVID+ group, clinical variables such as Oxygen Saturation at hospital admission (SO2%), mean of Oxygen Saturation during hospitalization and number of days in COVID ward were collected (see Table 1).

**TABLE 1:** Demographics and clinical data COVID+ group.

	Mean (ds)
Age	58.41 (10.00)
Gender	17 M/12 F
Education	11.07 (3.77)
Days in COVID ward	12.28 (5.35)
SO2% at admission	92.53 (3.25)
Mean SO2% during hospitalization	95.97 (0.93)
Mean P/F	234.78 (62.92)

We enrolled 29 patients (COVID+ group; M/F: 17/12; age 58.41 ± 10.00 years; education 11.07 ± 3.77 years, left handers 3) who agreed to participate in the study. Among the 16 patients who declined to participate, the main reasons were rejection (*N* = 10, with no motivation) and difficulties in having a stable connection for the TNP (*N* = 6); no significant differences in demographics were found between the group of enrolled patients and people who did not accept to take part in the study.

We enrolled 29 healthy controls (COVID−) who were never diagnosed with SARS-CoV-2 infections with a 1:1 match by age, education, and gender. The study design and protocol were approved by the local Ethics Committee (“ComitatoEtico di Brescia”) and written informed consent was obtained from all participants according to the Helsinki Declaration of 1967. A complete explanation of the study protocol was provided, and subjects could withdraw their participation at any point in the study.

### Procedure

After evaluating clinical records of patients dismissed from the COVID ward, we contacted the patients who matched the criteria, explained the study protocol and aims, and asked for their willing to participate. If the patient was available, a video call was scheduled for his/her most convenient time to perform the TNP evaluation.

The same procedure (phone contact and scheduled video call) was used for enrolling and evaluating the participants of the control group.

Cognitive and psychological assessment was performed *via* TNP by expert neuropsychologists for all the participants.

For the COVID+ group, evaluation was performed on average 20 days after hospital discharge to have the most recent possible cognitive profile related to the infection. A short interview with the caregiver was performed when available.

### Neuropsychological Evaluation

Both groups performed a cognitive and neuropsychiatric assessment. Cognitive and psychological data were collected *via* TNP by expert neuropsychologists. Patients and healthy controls underwent a battery of neuropsychological tests for evaluating several cognitive domains. We performed: Mini-Mental State Examination (MMSE) (Measso et al., 1993), Rey Auditory Verbal Learning Test (RAVLT) (Carlesimo et al., 1996), Colored Progressive Matrices 47 (CPM47) (Basso et al., 1987), Clock Drawing Test (CDT) (Mondini et al., 2011), The phonemic/semantic and alternate fluency test (Costa et al., 2014), Digit Span Forward and Digit Span Backward (Monaco et al., 2013). Moreover, we collected measures of mood and anxiety traits using the State-Trait Anxiety Inventory (STAI) (Spielberger, 1989) and the Beck Depression Inventory II (BDI-II) (Beck et al., 1961). Neuropsychological scores were adjusted by age, sex, and education according to normative data from the Italian population. Based on normative data, we created dichotomous variables (normal-pathological) for each test.

In the COVID+ group, we collected information from caregivers about patients’ premorbid status assessed through interviews with the patients’ caregivers. Caregivers were also asked to provide an explicit judgment regarding the patients’ mental functioning on a 5-point Likert scale, ranging from 1 (no symptoms) to 5 (symptoms always present), for the following cognitive domains: memory, language comprehension and production, attention, anxiety, and depression. For each domain, we asked to score the mental functioning before the SARS-CoV-2 infection and at present moment. Dichotomous values (change vs. no-change) were then elaborated for the questionnaire administered to the caregiver for each domain.

The current behavioral status was assessed through the Frontal Behavioral Inventory (FBI) with patients’ caregivers (Kertesz et al., 1997).

### Statistical Analysis

Statistical analysis was performed using SPSS Statistics 20.0 (SPSS Software, Chicago, IL, United States). Descriptive values of demographic and clinical data were presented as mean ± standard deviation (see Table 1). The significance level was set at 0.05.

Comparisons between groups on neuropsychological test results and behavioral data were conducted using parametric (*t*-test for repeated measures) or non-parametric (Mann–Withney *U*-test; Chi-square goodness of fit) tests according to data distribution. Shapiro–Wilk test was used to verify the homogeneity of variances.

Within COVID+ group, we also evaluated the correlation (two-tailed Pearson’s r correlation) between the cognitive and behavioral assessment scores and clinical variables collected.

## Results

**FIGURE 1:**
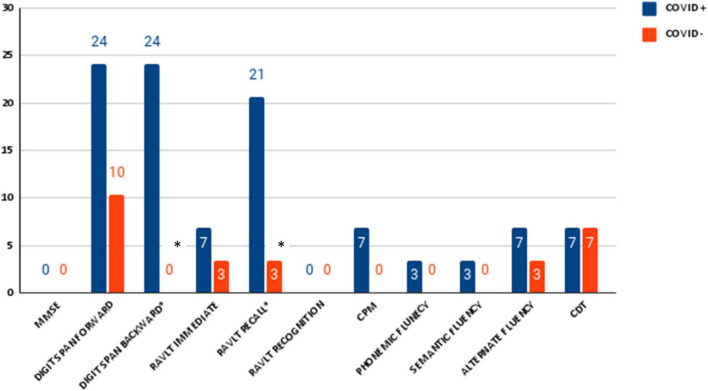
Percentage of number of pathological neuropsychological test in patients with SARS-CoV-2 and control group. Significance is indicated with *.

**FIGURE 2:**
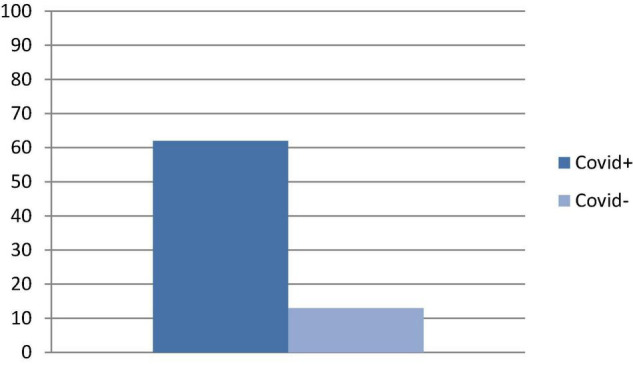
Percentage of number of people with at least one pathological score in NPS tests.

The two groups were matched for age, gender, and education, therefore no difference was expected between those variables. As for the cognitive assessment, we found significant differences between groups in the RAVLT scores (learning, recall, and recognition) (see Table 2 for details). Chi square on dichotomous values (normal/pathological) showed a significant difference between groups in Digit backward test (pathological 7/29 COVID+ vs. 0/29 COVID−; *p* = 0.005) (see Figure 1 for details). Overall, the number of people with at least one pathological score was higher in the COVID+ group than in controls (18/29 COVID+ vs. 4/29 COVID−; *p* = 0.000) (see Figure 2 for details). We did not find any difference in the number of people with depression (BDI-II) or anxiety (STAI scales) between groups when looking at dichotomous values. However, trait anxiety scale (STAI-II) score was significantly higher in the COVID− group (*p* = 0.002).

**TABLE 2:** Comparisons of the cognitive and psychological assessment scores between COVID+ and COVID− groups.

	COVID+ (*N* = 29)	COVID− (*N* = 29)	*p*
**Cognitive assessment**			
MMSE	28.9 (1.37)	29.21 (0.68)	n.s.
Raven CPM	29.48 (4.72)	30.53 (3.48)	n.s.
Digit forward	5.34 (0.97)	5.69 (1.14)	n.s.
Digit backward	4.24 (1.21)	4.76 (0.87)	n.s.
RAVLT immediate	42.55 (10.44)	47.9 (8.29)	**0.035**
RAVLT recall	8.79 (3.13)	10.38 (2.19)	**0.03**
RAVL recognition	13.69 (1.47)	14.52 (0.63)	**0.007**
Phonemic fluencies	32.45 (10.19)	35 (8.51)	n.s.
Semantic fluencies	46.59 (7.65)	49.38 (7.58)	n.s.
Alternate fluencies	31.14 (11.98)	31.59 (10.26)	n.s.
CDT	8.81 (2.22)	9. 38 (1.23)	n.s.
**Psychological assessment**			
BDI II	9.03 (8.6)	8.52 (4.96)	n.s.
STAI Y-1	35.86 (9.63)	38.41 (7.05)	n.s.
STAI Y-2	32.69 (7.66)	39.14 (7.67)	**0.002**

*Mean, standard deviation, and P is reported for t-test statistics. Significance value are highligted in bold.*

**FIGURE 3:**
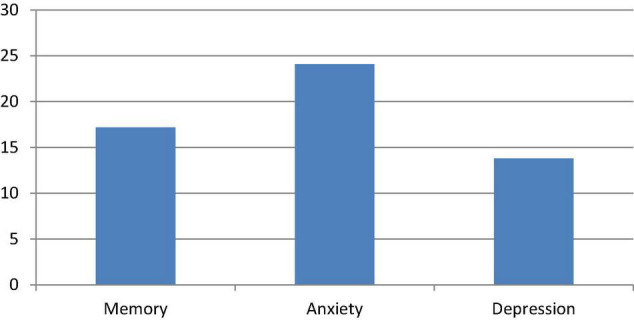
Percentage in worsening in COVID+ group referred by the caregiver.

Caregivers’ interviews were conducted in COVID+ group only and revealed worsening in memory in 17.2% of patients, an increase of anxiety in 24.1%, and a worsening mood in 13.8% of one’s compared to their status before infection (see Figure 3 for details).

## Discussion

To the best of our knowledge, this is one of the few studies aimed at exploring cognitive and neuropsychiatric sequelae of COVID-19 by comparing neuropsychological assessment in previously hospitalized patients who did not need ICU access and matched control subjects who were never diagnosed with COVID-19.

We compared the performances in some cognitive tasks and mood/anxiety measures between patients dismissed from the COVID ward and healthy controls. As for the cognitive performances, learning and memory abilities were significantly more impaired in the COVID+ group. In agreement with these findings, the caregivers have the subjective impression of a worsening in memory but not attentive or language abilities.

We did not find significant differences between groups in depression symptoms; only the state anxiety scale was significantly higher in the control group.

### Cognition

We found that the number of people with at least one pathological score was higher in patients with SARS-CoV-2 infection than in controls. These findings reflect previous research showing cognitive deficits in COVID-19 patients such as impairments in memory (Almeria et al., 2020; Woo et al., 2020; Alemanno et al., 2021; Jaywant et al., 2021; Negrini et al., 2021; Stracciari et al., 2021; Yesilkaya et al., 2021), executive functions (Almeria et al., 2020; Alemanno et al., 2021; Beaud et al., 2021; Jaywant et al., 2021; Negrini et al., 2021; Stracciari et al., 2021; Yesilkaya et al., 2021), attention (Almeria et al., 2020; Woo et al., 2020; Graham et al., 2021; Jaywant et al., 2021; Negrini et al., 2021; Stracciari et al., 2021; Yesilkaya et al., 2021), and working memory (Almeria et al., 2020; Graham et al., 2021; Jaywant et al., 2021; Negrini et al., 2021; Stracciari et al., 2021).

We found that patients with COVID-19 have worse performance than healthy controls in memory abilities (learning, recall, and recognition). Furthermore, when using normative data taking gender, age, and education into account, the percentage of pathological tests in those domains was significantly higher in COVID+ then in COVID−.

In agreement with previous studies on other acute respiratory syndromes (SARDS) (Hopkins et al., 1999, 2005, 2006; Wilcox et al., 2013) and Obstructive Sleep Apnea Syndrome (OSAS) (Wallace and Bucks, 2013), in our COVID+ patients, we found memory impairment, in particular in free and delayed verbal recall and recognition. It is worth mentioning that studies on mice infected with influenza viruses have shown a change in the morphology and functionality of the hippocampus (Hosseini et al., 2018) resulting in deficits in learning. In a recent commentary Yesilkaya and Balcioglu (2020), pointed out that neuropsychiatric manifestation of SARS-CoV-2 might be associated with the involvement of both neuroimmune response and direct viral transmission.

Damage is shown also by MRI study. Douaud et al. (2021) compared cortical thickness and volume index in patients before and after SARS-CoV-2 infection showing a significant reduction in cortical volume and thickness in areas related to memory tasks in the left hemisphere (i.e., lateral orbitofrontal cortex, the dorsal insula, and the parahippocampal gyrus). The left anterior cingulate cortex, the left supramarginal gyrus, and the right temporal pole were also affected.

Interestingly, the left parahippocampal gyrus, in particular the perirhinal cortex, is directly connected to the piriform cortex and entorhinal cortex, which are both part of the primary olfactory cortex, the principal pathway of SARS-CoV-2 infection. Moreover, Douaud and colleagues found greater gray matter loss in COVID-19 patients with longer hospitalization.

Greater degeneration in the left than right olfactory cortex and decrement of odor detection sensitivity have been described also in patients with AD. Therefore, we could speculate that our findings showing a memory impairment in COVID+, may be due to an increased sensitivity of the hippocampus to the infection (Wilson M. G. et al., 2020).

These results are confirmed by caregiver impressions through compilation of questionnaires (5-point Likert scale) which evaluated changes in cognitive and neuropsychiatric areas. Caregivers reported a subjective worsening only in memory among other cognitive domains.

Moreover, Chi-square on dichotomous values showed a significant difference between groups in the Digit backward test. Thirty percent of patients with COVID-19 have a pathological score compared to none in the control group. These findings are in agreement with previous studies showing an impairment in working memory abilities in COVID+ patient (Almeria et al., 2020; Graham et al., 2021; Jaywant et al., 2021; Negrini et al., 2021; Stracciari et al., 2021). Again, this data may be due to the neuroinvasive nature of the virus. In fact, similar to many respiratory viruses, in COVID-19 the most commonly reported entry is through the olfactory bulb which is directly linked with the frontal cortex (Mirfazeli et al., 2020; Pantelis et al., 2020; Kumar and Nayar, 2021), that is the principal cerebral region involved in attention and working memory abilities (Zhou et al., 2020).

### Psychological Impact

In our sample, worsening mood and anxiety have been described by some caregivers. However, the state anxiety scale was significantly higher in the control group. This data is not in line with some findings about a worsening in mood and anxiety in patients with COVID-19 (Rogers et al., 2020). This may be due to the small number of subjects enrolled to date or to a psychological reaction to the threat to life they just got away from. The pandemic and the stay-at-home orders have also led to a change in lifestyle, such as sleep quality, physical activity, and eating behavior (Flanagan et al., 2021). As confirmation, several studies (AdriánPriego-Parra et al., 2020; Gao et al., 2020; Petzold et al., 2020) show an increase of anxiety and depression symptoms during COVID-19 even in healthy people that consider SARS-CoV-2 a real threat to their health. Moreover, they spent a greater amount of time ruminating about COVID-19 in everyday life and an increased fear of becoming infected with COVID-19 (Petzold et al., 2020). Finally, because CoV tends to persist in the CNS (Yesilkaya and Balcioglu, 2020), long-term follow-up and neuropsychiatric monitoring should be performed.

### Strengths and Limitation

Our study has some limitations that should be taken into consideration. First of all, the sample size was relatively small: only 29 patients and 29 healthy controls were included; furthermore, due to the explorative nature of the study, we did not perform a correction for multiple comparisons to avoid the risk of increasing type II errors. Another limitation is that we cannot completely exclude that some subjects in the control group may have had SARS-CoV-2 infection without any symptoms; however, since we found significant differences between groups, it seems that this eventual inclusion of asymptomatic controls did not affect the validity of our findings.

Another point may be that neuropsychological assessments delivered *via* telehealth have highlighted cognitive impairments in COVID-19 patients, we have not investigated some cognitive domain evaluable only with face-to-face assessment or paper-pencil test.

Finally, results would have been better explained if we could have a neuroimaging correlate of the findings.

Strengths of the study are a well-matched control group, a very selected sample (no ICU access, relatively young age, no previously known neurological illnesses) and the fact we assessed COVID+ patients after a 2–3 weeks period post-discharge which allowed them to recover and to settle in once back home. This last point is particularly important since the deficits we found are less likely due to the hospitalization *per se*.

Finally, although telehealth does not allow a complete and deep cognitive evaluation (some tests need to be performed in the presence), its usefulness and reliability have been demonstrated (Marra et al., 2020; Sozzi et al., 2020) and it makes it easier to evaluate patients who would not be prone to come back to the clinic due to pandemic situation or their health issues.

Finally, for those patients who continue to have cognitive dysfunctions even after some time, it may be useful a cognitive rehabilitation with exercises for memory and executive functions to improve these abilities.

## Conclusion

Our results confirm that SARS-CoV-2 infection may cause cognitive deficits involving memory and executive functions in patients hospitalized without acceding to ICU. Additional studies with a larger sample size and follow-up are needed to further confirm the results and evaluate the progression, stability, or remission of the deficits. To date, in fact, it is still unknown whether patients would spontaneously recover or if those cognitive deficits could progress. Teleneuropsychology may be a useful tool to further investigate this topic.

## Data Availability Statement

The raw data supporting the conclusions of this article will be made available by the authors, without undue reservation.

## Ethics Statement

The studies involving human participants were reviewed and approved by Comitato Etico di Brescia. The patients/participants provided their written informed consent to participate in this study.

## Author Contributions

VC wrote the text, conceived and designed the experiments, provided substantial contributions to discussion, and edited the manuscript before submission. ADL wrote the text, conceived and designed the experiments, performed the experiments, provided substantial contributions to discussion, generated the figures, researched data for the manuscript, and edited the manuscript before submission. CS conceived and designed the experiments, analyzed the data, wrote the text, provided substantial contributions to discussion, and edited the manuscript before submission. AG researched data for the manuscript. MC conceived and designed the experiments and provided substantial contributions to discussion. All authors contributed to the article and approved the submitted version.

## Conflict of Interest

The authors declare that the research was conducted in the absence of any commercial or financial relationships that could be construed as a potential conflict of interest.

## Publisher’s Note

All claims expressed in this article are solely those of the authors and do not necessarily represent those of their affiliated organizations, or those of the publisher, the editors and the reviewers. Any product that may be evaluated in this article, or claim that may be made by its manufacturer, is not guaranteed or endorsed by the publisher.
